# C18:1 Methyl Ester Metathesis in [bmim][X] Type Ionic Liquids

**DOI:** 10.3390/ijms10115020

**Published:** 2009-11-18

**Authors:** Priya A. Thomas, Bassie B. Marvey

**Affiliations:** Department of Chemistry, North-West University, P/Bag X2046, Mafikeng, 2735, South Africa; E-Mail: priyaaregi@gmail.com (P.A.T.)

**Keywords:** metathesis, methyl oleate, [bmim][X], RuCl_2_(PCy_3_)(L)(=CHPh)

## Abstract

The efficacy of [bmim][X] ionic liquids (ILs) (X = PF_6_^−^, BF_4_^−^ and NTf_2_^−^) as reaction media for methyl oleate metathesis was compared with that of conventional organic solvents (PhCl, PhMe, DCM and DCE) using the well-defined first and second generation Grubbs precatalysts, RuCl_2_(PCy_3_)(L)(=CHPh) (L = PCy_3_ or H_2_IMes). Best catalytic performance, with excellent selectivity (>98%) at moderate reaction temperatures, was achieved in [bmim][X] ILs compared to conventional solvents. The effects of anion, reaction temperature, solvent polarity, solvent viscosity, and ligand-anion interaction on the reaction are also addressed.

## 1. Introduction

Room temperature ionic liquids (RTILs) have recently attracted considerable attention as potential alternatives to conventional organic solvents due to several of their unique physicochemical properties such as high polarity, non-volatility, non-flamability and immiscibility with water and/or organic solvents [1–6]. RTILs also provide excellent solvent media in many homogeneously catalyzed reactions such as olefin hydrogenation, hydroformylation, oligomerization and Pd mediated carbon-carbon coupling reactions and allow for simple recycling and reuse of transition metal catalysts [7–9]. The discovery of the well-defined ruthenium catalysts by Grubbs and coworkers has also advanced olefin metathesis as a powerful tool in organic synthesis [10,11]. However, the use of these catalysts in chemical industry is restricted by the fact that they are non-recyclable and their separation from the product stream still poses a serious challenge. Furthermore, most of the work on RTILs reported in the literature is based on ring closing metathesis (RCM) [12–17] and ring opening metathesis (ROM) [18]. Self- metathesis (SM) and cross-metathesis (CM) reactions in RTILs remain almost unexplored and very few works have been published [19–21].

**Scheme 1.**
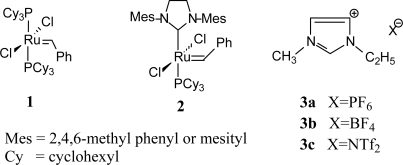
Grubbs precatalysts and imidazolium ILs.

The present study aims to investigate the self-metathesis of methyl oleate in [bmim][X] type ionic liquids using the well-defined first and second generation Grubbs precatalysts, RuCl_2_(PCy_3_)_2_(=CHPh) (**1**) and RuCl_2_(PCy_3_)(H_2_IMes)(=CHPh) (**2**) (Scheme 1). In particular, the influence on the catalytic activity by the anion (X) and other reaction parameters was examined. The study further evaluates the metathesis activity in [bmim][X] ILs against the activity in conventional organic solvents (PhCl, PhMe, DCM and DCE).

Fatty acid esters derived from seed oils are an alternative source of chemicals, since their chemical structures are closely related to those of the hydrocarbons in crude oil. Seed oil and their derivatives have recently attracted much attention due to their renewable supply, low cost, versatility and their green chemistry. Fatty acid monoesters, like methyl oleate, are usually derived from transesterification of seed oil with lower alcohols, yielding glycerol as the by-product [22]. Self and cross metathesis of fatty acid esters would result in the formation of monomers, dimers and α-olefins with interesting applications in polymer, pharmaceutical and petrochemical industries [23,24].

## 2. Results and Discussion

### 2.1. Self-Metathesis in [bmim][X] Type Ionic Liquids

**Scheme 2.**
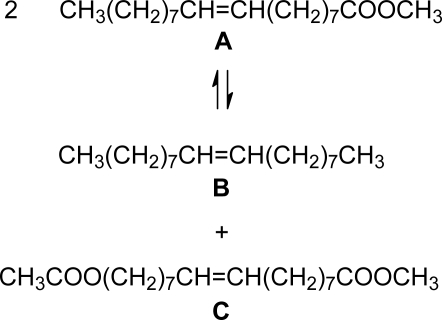
Primary metathesis products from self-metathesis of methyl oleate.

The self-metathesis of methyl oleate in [bmim][X] type RTILs was carried out in presence of Grubbs precatalysts **1** and **2**. Different anions (X = hexafluorophosphate (PF_6_^−^), tetraflouroborate (BF_4_^−^) and bis (trifluoromethylsulphonyl)imide (NTf_2_^−^)) in conjunction with a common cation, 1-butyl-3-methylimidazolium (bmim) were tested for their suitability as reaction media. Table 1 presents some of the properties of [bmim][PF_6_], [bmim][BF_4_] and [bmim][NTf_2_]. PF_6_^−^ and BF_4_^−^ anions are neutral, weakly coordinating and form highly viscous imidazolium ILs while PF_6_^−^ and NTf_2_^−^ form hydrophobic ILs with NTf_2_^−^ forming a less viscous imidazolium IL [25]. When mixed with methyl oleate all the ILs formed a biphasic system. Two primary metathesis products (PMPs) were obtained in the metathesis of methyl oleate (**A**), namely, octadec-9-ene (**B**) and dimethyl octadec-9-ene-1,18-dioate (**C**), as illustrated in Scheme 2 [26].

**Figure 1.**
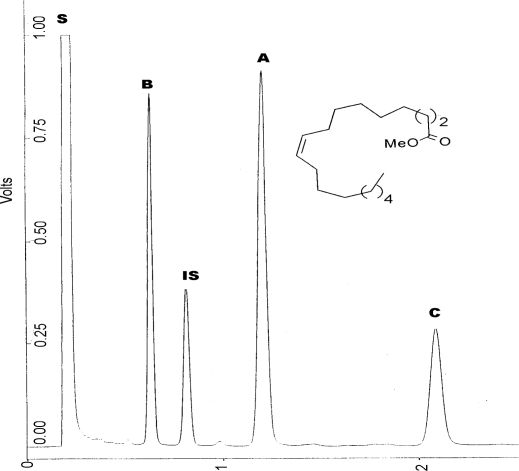
Typical gas chromatogram resulting from the metathesis of methyl oleate.

Figure 1 shows a typical gas chromatogram resulting from the metathesis of methyl oleate with nonadecane as internal standard (**IS**).

#### 2.1.1. Influence of RTIL Anion [X]

**Figure 2.**
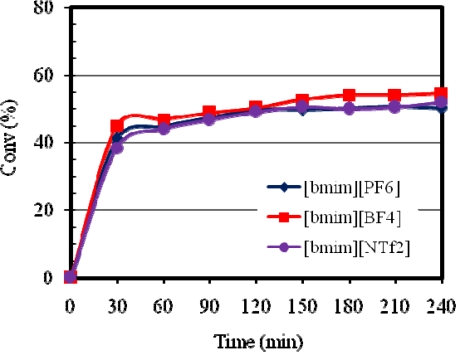
Influence of [bmim][X] type ILs on the activity of **1** at 20 °C.

**Figure 3.**
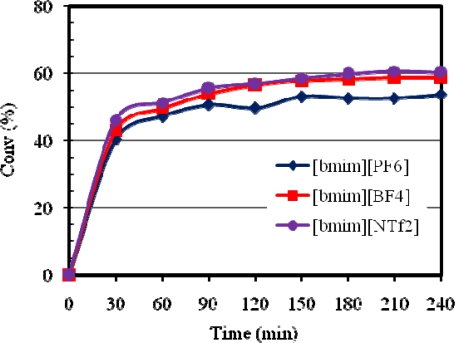
Influence of [bmim][X] type ILs on the activity of **2** at 20 °C.

Anions play a significant role in determining the properties of ILs. For example, [bmim][PF_6_] is immiscible with water, whereas, [bmim][BF_4_] is soluble in water. Furthermore, the anions determine viscosity, density, hydrophobicity and solvation of ILs [27]. The effect of varying the anion counterpart in [bmim][X] type RTILs on the metathesis of methyl oleate using Grubbs catalysts has been studied. Figure 2 shows the influence of anion on the catalytic activity of **1**. The metathesis activity showed a decreasing trend with catalyst **1** according to the solvent/anion order BF_4_^−^ > NTf_2_^−^ ~ PF_6_^−^. For catalyst **2** the trend in metathesis activity decreased in the order NTf_2_^−^ ~ BF_4_^−^ > PF_6_^−^ as illustrated in Figure 3.

Clearly only a small difference in catalytic activity occurred in different ILs for both **1** and **2**. The effect on solvent efficacy and the catalytic activity upon changing the ligand L on the catalyst was evident from a change in the activity trend as PCy_3_ replaced H_2_IMes. However, no simple correlation could be deduced between solvent polarity and catalytic activity like it was the case for conventional organic solvents [26]. On the other hand it could be that the coordination ability of the anion to the catalyst was important in determining the activity pattern as observed [28].

#### 2.1.2. Influence of Reaction Temperature

**Scheme 3.**
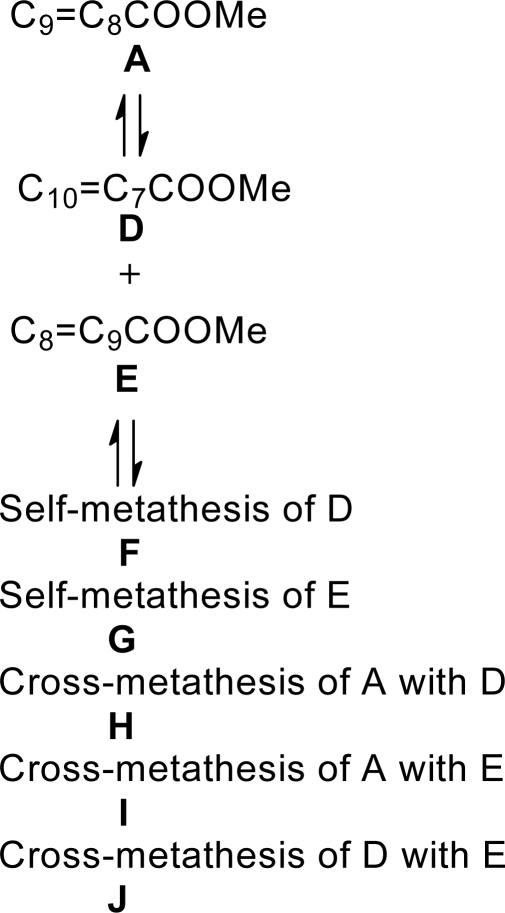
SMPs resulting from the metathesis of methyl oleate.

Table 2 summarizes the activity of **1** and **2** in **3a–3c** at different reaction temperatures in a closed system. Increasing the reaction temperature from 20–100 °C resulted in a rate enhancement with optimum reaction temperature reached at almost 80 °C in **3a**. Excellent selectivity (>98%) was achieved at moderate reaction temperatures (≤60 °C) but decreased at higher temperatures with the formation of secondary metathesis products (SMPs) (F to J) shown in Scheme 3 [23]. The formation of SMPs at high reaction temperatures was evidence of double bond isomerization (A to D and E) occurring.

At 60 °C, the best catalytic performance occurred in **3b** with **2** generally displaying superior activity than **1** in all the runs. However, **2** suffered a significant loss in selectivity at temperatures >60 °C compared to **1**, with the result that comparatively higher yields of SMPs were obtained. A radical change in the activity trend from NTf_2_^−^ > BF_4_^−^ > PF_6_^−^ at 20 °C to BF_4_^−^ > PF_6_^−^ > NTf_2_^−^ at 60 °C in the case of **2** was quite remarkable, especially for NTf_2_^−^ which showed more sensitivity towards a change in reaction temperature. It appears in this instance, given the inverse relationship that exists between temperature and viscosity of ILs [25], that the lowering in the viscosity of [bmim][NTf_2_] through an increase in reaction temperature might have compromised its solvent efficacy and performance. Such an effect was, however, not so much pronounced for BF_4_^−^ and PF_6_^−^ whose viscosities at elevated temperatures remained relatively high to that of NTf_2_^−^ given their extreme high viscosities at room temperature. If this was to be true for NTf_2_^−^, then BF_4_^−^ and PF_6_^−^ would be seen as more suited for high temperature reactions and NTf_2_^−^ for reactions at low temperatures.

For MO/Ru molar ratio of 10,000, TONs of 2,300 and 6,200 were obtained at 60 °C in **3a** for **1** and **2**, respectively. The difference in TONs could be attributed to the short lifetime and poor thermal stability of **1** compared to **2** [29,30]. In spite of significantly enhanced activity of **2** relative to **1**, catalyst **2** displayed poor selectivity, especially at high reaction temperatures. High selectivities and TONs are some of the important indicators if the process is to find industrial application.

### 2.2. [bmim][X] ILs vs. Conventional Organic Solvents

Self-metathesis of MO in organic solvents was carried out in presence of **1** and **2** and the results are summarized in Table 3. The solvents used for this study were DCM, DCE, PhMe and PhCl. The best catalytic performance among the conventional solvents occurred in DCM and the lowest in PhMe. The activity of **1** and **2** were found to increase in the order PhMe < PhCl < DCE ~ DCM in accordance to an increase in solvent polarity. Selectivity towards PMPs was 100% in all the organic solvents. These results are in agreement with the work done by Buchowicz and Mol [31] and Marvey *et al*. [26].

**Figure 4.**
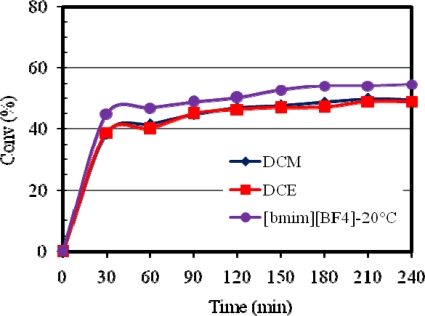
Activity of **1** in [bmim][BF_4_], DCM and DCE at 20 °C.

**Figure 5.**
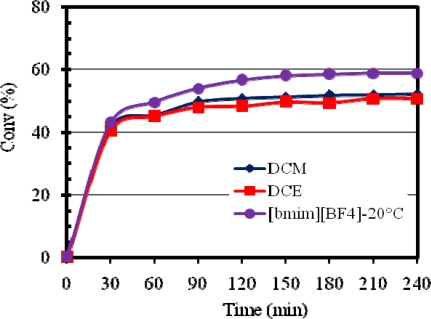
Activity of **2** in [bmim][BF_4_], DCM and DCE at 20 °C.

Compared to [bmim][X] ILs, the conventional organic solvents were less efficient in that relatively lower methyl oleate conversions were obtained for both **1** and **2**. Figures 4 and 5 compare the activities of **1** and **2**, respectively, in [bmim][BF_4_], DCM and DCE.

From the results obtained, it is clear that a significant rate enhancement occurred in [bmim][BF_4_] as opposed to the best of the conventional solvents, namely, DCM and DCE. A further substantial rate enhancement occurred in [bmim][BF_4_] at a higher reaction temperature (60 °C). Therefore, [bmim][X] ILs provide excellent reaction media for methyl oleate metathesis and can be used as convenient substitutes for conventional organic solvents due to their “green” characteristics and possibilities for easy product separation and catalyst recycling [9].

## 3. Experimental Section

### 3.1. Materials and Apparatus

Chlorobenzene (PhCl), dichloromethane (DCM), 1,2-dichloroethane (DCE), toluene (PhMe), 1-butyl-3-methylimidazolium hexafluorophosphate ([bmim][PF_6_]), 1-butyl-3-methylimidazolium tetra-fluoroborate ([bmim][BF_4_]), 1-butyl-3-methylimidazolium bis (trifluoromethylsulphonyl)imide ([bmim][NTf_2_]) were all reagent grade from Sigma-Aldrich. Methyl oleate (≥ 99%) was obtained from Sigma-Aldrich and was treated with activated alumina and stored under N_2_ at a subzero temperature. Ethyl vinyl ether was purchased from Fluka. Nonadecane purchased from Fluka was used as the internal standard (**IS**). Grubbs precatalysts **1** and **2** were stored under N_2_ and used as purchased from Sigma-Aldrich. Chromatograms were obtained using Varian Star 3400 *CX* GC equipped with a DB-624 capillary column (J&W Scientific, 30 m × 0.53 mm) and a flame ionization detector (FID). The oven temperature was held at 200 °C and then increased to 270 °C at a rate of 20 °C min^−1^. The injector temperature was set at 270 °C and the detector temperature at 300 °C with N_2_ as carrier gas.

### 3.2. Metathesis Experiments

All the reactions were performed under a N_2_ atmosphere in a glass reactor fitted with a thermometer and a rubber septum. For the reaction in organic solvents, 12.4 mg of **1** (0.015 mmol) or **2** (0.014 mmol) was dissolved in 2.0 mL of organic solvent followed by 0.1 g of internal standard and 0.5 mL substrate. For the reaction in RTILs, 0.5 mL of the substrate was added to 1 mL of ionic liquid and stirred for 10 min to attain the reaction temperature. An internal standard (0.05 g) was added followed by the addition of 12.4 mg of **1** or **2**. Both catalysts were soluble in ILs with the substrate forming a biphasic mixture with ILs. Samples were withdrawn by a syringe at regular time intervals for up to 5 hours. The reaction was terminated by immediately quenching with a few drops of ethyl vinyl ether [31]. The quenched sample was diluted with solvent (**S**) and analyzed by GC. The following formulas were used in the calculations:

Conversion(%) = n0−ntn0 x 100

where *n_0_* = number of moles of substrate at the beginning of the reaction. *n_t_* = number of moles of substrate after time *t*.

$$\text{Selectivity}(\%)\;=\;\frac{\text{yield}\;\text{of}\;PMPs}{\sum \text{yield}\;of\;\text{products}}\;x\;100$$

*TON = MO/Ru ratio x substrate conversion*; TON is the number of moles of substrate that a mole of catalyst can convert before becoming inactivated.

## 4. Conclusions

The efficacy of [bmim][X] ILs as reaction media in methyl oleate metathesis was evaluated and the effects of anion, reaction temperature, solvent polarity, viscosity and ligand-anion interaction on the reaction have been addressed. The nature of the anion in [bmim][X] ILs proved to have a slight influence on catalytic performance, suggesting that only a small degree in rate enhancement could be expected from the variation of the anion. The results obtained further indicate that reaction temperature has a significant effect on solvent efficacy with anions displaying varied sensitivities upon a change in ligand L and reaction temperature. While excellent selectivity is achieved at a moderate reaction temperature, a loss in selectivity occurs at higher reaction temperatures with the formation of SMPs and is more pronounced for L = H_2_IMes. Indeed [bmim][X] ILs outperformed conventional solvents as reaction media for the ruthenium-catalysed self-metathesis of fatty acid methyl esters and have demonstrated their potential as excellent substitutes to these solvents with an added advantage that they meet greener character requirements and allow for easy catalyst separation and recycling.

## Figures and Tables

**Table 1.** Polarity (E_T_^N^), solubility and viscosity of [bmim][X] ILs [25].

X	$E_{T}^{N}$	Solubility in H_2_O	Viscosity (cP)(25 °C)

PF_6_	0.667	Insoluble	207
BF_4_	0.673	Soluble	233
NTf_2_	0.642	Insoluble	52

**Table 2.** Activity of **1** and **2** in **3a–3c** after 4 hour reaction time.

**RTIL**	**Catalyst**	**Temp. (°C)**	**Conv (%)**	**Selec**^[a]^**(%)**	**TON**

**3a**	**1**	20	50.1	100	50.1
	**2**		52.9	100	52.8
	**1**	40	57.1	100	57.1
	**2**		64.9	90	64.9
	**1**	60	59.4	99	59.4
	**2**		74.9	81	74.9
	**1**	80	61.6	92	61.6
	**2**		79.0	60	79.0
	**1**	100	62.0	92	62.0
	**2**		79.1	47	79.1
**3b**	**1**	20	54.4	100	54.4
	**2**		58.8	100	58.8
	**1**	60	61.1	95	61.1
	**2**		78.2	87	78.2
**3c**	**1**	20	51.2	100	51.2
	**2**		60.5	99	60.5
	**1**	60	60.5	99	60.5
	**2**		72.7	72	72.7
3a*	**1**	60	23.0	88	2300
	**2**		62.0	85	6200

MO/Ru ratio = 100,

^[a]^ selectivity towards PMPs.

* MO/Ru molar ratio = 10,000.

**Table 3.** Activity of **1** and **2** in conventional organic solvents.

**Entry**	**Solvent**	**Catalyst**	**Conv (%)**	**Selec (%)**

1	PhCl	**1**	47.1	100
		**2**	50.6	100
2	PhMe	**1**	45.0	100
		**2**	48.0	100
3	DCM	**1**	49.0	100
		**2**	51.3	100
4	DCE	**1**	48.9	100
		**2**	50.7	100

MO/Ru ratio 100, 20 °C, 4 h.
